# The effect of time-varying left ventricular ejection fraction on clinical outcome in critically ill COVID-19 patients

**DOI:** 10.1016/j.aicoj.2026.100171

**Published:** 2026-09-29

**Authors:** M.S. Chew, S. Jansson, M. Jozwiak, Z. Hassanzadeh, H. Didriksson, C. Jonsson, H. Andersson, M. Åström Aneq, M. Slama, A. Vieillard Baron, P. Vignon, H. Zhang, S. Huang

**Affiliations:** a Department of Clinical Science, Intervention and Technology, Karolinska Institutet, Stockholm, Sweden; b Department of Anesthesia and Intensive care, Linköping University Hospital, Linköping, Sweden; c Department of Clinical Physiology, Linköping University Hospital, Linköping, Sweden; d Anesthesia and Intensive care, Västerviks sjukhus, Region Kalmar län, Sweden; e Service de Médecine Intensive Réanimation, CHU de Nice Hôpital Archet 1, Nice, France; f UR2CA, Unité de Recherche Clinique Côte d'Azur, Université Côte d'Azur, Nice, France; g Intensive Care Unit, Amiens-Picardie University Hospital, Amiens, France; h Medical and Surgical ICU, University Hospital Ambroise Pare, AP-HP, Boulogne-Billancourt, France; i Medical Surgical Intensive Care Unit, Limoges University Hospital, Limoges, France; j Intensive Care Medicine, Nepean Hospital, NBMLHD, The University of Sydney, Sydney, Australia; k Malmö University, Malmö, Sweden

**Keywords:** Critical care, Echocardiography, COVID-19, Left ventricular function, ICU mortality

## Abstract

**Background:**

Although right ventricular (RV) dysfunction and acute cor pulmonale are associated with adverse outcomes in critically ill patients with COVID-19, the prognostic significance of left ventricular (LV) systolic dysfunction remains insufficiently characterized. In particular, dynamic changes in cardiac function during intensive care has seldom been studied. We therefore investigated the association between time-varying LV systolic function, assessed by LV ejection fraction (LVEF) and intensive care unit (ICU) mortality in patients with COVID-19, taking into account right ventricular function and forward flow.

**Methods:**

This retrospective observational cohort study combined three ICU data repositories comprising 808 patients with 1614 echocardiographic examinations. Time-varying LV systolic function (LVEF ≤ 50%, 51–70%, >70%) was the primary exposure, and ICU mortality was the outcome. Multivariable Cox models with time-varying covariates, including, acute cor pulmonale, left ventricular outflow tract velocity time integral and key clinical characteristics were used to assess associations between LV systolic function phenotypes and outcome.

**Results:**

At baseline, 27% of patients had abnormal LV systolic function (LVEF ≤ 50%) and 17% had hyperdynamic LV function (LVEF > 70%). The proportion of patients with normal LV systolic function increased over time. Abnormal LV systolic function was most prevalent at the first echocardiographic examination (27%) and least prevalent at the fifth examination (17%), whereas hyperdynamic LV function became more frequent during ICU stay, peaking at the 4th echocardiographic examination. Abnormal (aHR; 1.174, 95% CI: 0.901–1.531) or hyperdynamic (aHR: 1.041, 95% CI: 0.785–1.379) LV systolic dysfunction was not independently associated with ICU mortality.

**Conclusion:**

In critically ill patients with COVID-19, LV systolic dysfunction, including both abnormal and hyperdynamic LV phenotypes, were common and dynamic during ICU stay. LV systolic dysfunction was not independently associated with ICU mortality when modelled longitudinally and adjusted for RV dysfunction, forward flow and other clinical covariates. These findings suggest that the prognostic value LVEF phenotypes in severe COVID-19 may be limited. Further studies incorporating more sensitive measures of LV function and biomarkers of myocardial injury may be required to delineate subtle changes not captured by LVEF.

## Background

Cardiac dysfunction occurs commonly in Coronavirus disease 2019 (COVID-19) and may range from asymptomatic myocardial injury to fulminant myocarditis with cardiogenic shock [1]. Preexisting cardiovascular disease and cardiovascular risk factors predispose patients to more severe disease and mortality [2,3]. Right ventricular (RV) dysfunction, particularly right ventricular dilatation and acute cor pulmonale (ACP), are prognostically important for short term mortality [[4], [5], [6], [7], [8]].

In contrast, the prognostic significance of left ventricular (LV) systolic dysfunction remains less well investigated. The severe acute respiratory syndrome coronavirus 2 (SARS-CoV-2) is a cardiotropic virus and is associated with myocardial injury, with reported prevalences of LV dysfunction varying from 9% to 35% [[7], [8], [9], [10], [11], [12]].

In the EchoCOVID study of critically ill patients admitted to intensive care units (ICUs), the prevalence of moderate to severe RV systolic dysfunction increased in parallel with the severity of LV dysfunction [6]. In one of the few prospective, systematic studies of consecutive, hospitalized patients with COVID-19 both low left ventricular ejection fraction (LVEF) and increased RV end diastolic area (EDA) at baseline were associated with mortality [9]. A systematic review of echocardiographic studies in the critically ill demonstrated that although LVEF was within a normal range for a majority of patients, low LVEF and RV dilatation were associated with increased mortality [13]. These findings suggest a prognostic role for decreased LV function, and an interacting effect with RV function.

Reported incidences of left ventricular dysfunction vary greatly and there is conflicting data on its impact on outcome [[12], [13], [14]]. No study has examined the longitudinal effects of LV systolic function while simultaneously accounting for right ventricular function. Of note, no study has taken into account forward flow as a key determinant of clinical outcomes – which is particularly concerning since cardiac output may be maintained even when LVEF is reduced and vice versa, may be decreased when LVEF is increased. Previous studies have largely examined single snapshots of echocardiographic data, without accounting for potential temporal changes in ventricular function during the course of critical illness. To address these knowledge gaps, we investigated the time-varying association between LV systolic function and ICU mortality among critically ill COVID-19 patients, adjusting for right ventricular function, forward flow and relevant clinical characteristics.

## Methods

We used data from three different repositories; EchoCOVID, SweCOVID and Nice University hospital. All repositories consisted of adult patients (≥18 years) with confirmed SARS-CoV-2 infection. EchoCOVID is a retrospective longitudinal, observational study of patients admitted to 14 ICUs of tertiary teaching hospitals in 8 countries (NCT 04414410). Consecutive patients between 1 February 2020 and 26 December 2022, admitted to an ICU and who received at least one critical care echocardiography (CCE) in ICU, either a transthoracic (TTE) or transesophageal echocardiography (TEE), were included. SweCOVID included consecutive ICU admissions at Linköping University Hospital between 6 Mar 2020–31 Dec 2022. The Nice University Hospital database included consecutively admitted patients in two hospitals between Mar 2020 and Mar 2021. The latter 2 repositories included routine echocardiographic examinations on all patients within 72 h of ICU admission. Patients were included in the present study if LVEF was recorded on the first echocardiographic examination and ICU outcome and length of stay was documented (Fig. 1). All three databases have been approved for use in this study by relevant local ethics committees (EchoCOVID, CE SLRF 20-40; Nice, R04-0223313140420; SweCOVID, Dnr 2020-01884). We adhered to the STROBE guidelines for reporting on cohort studies (Supplementary material).

**Fig. 1:**
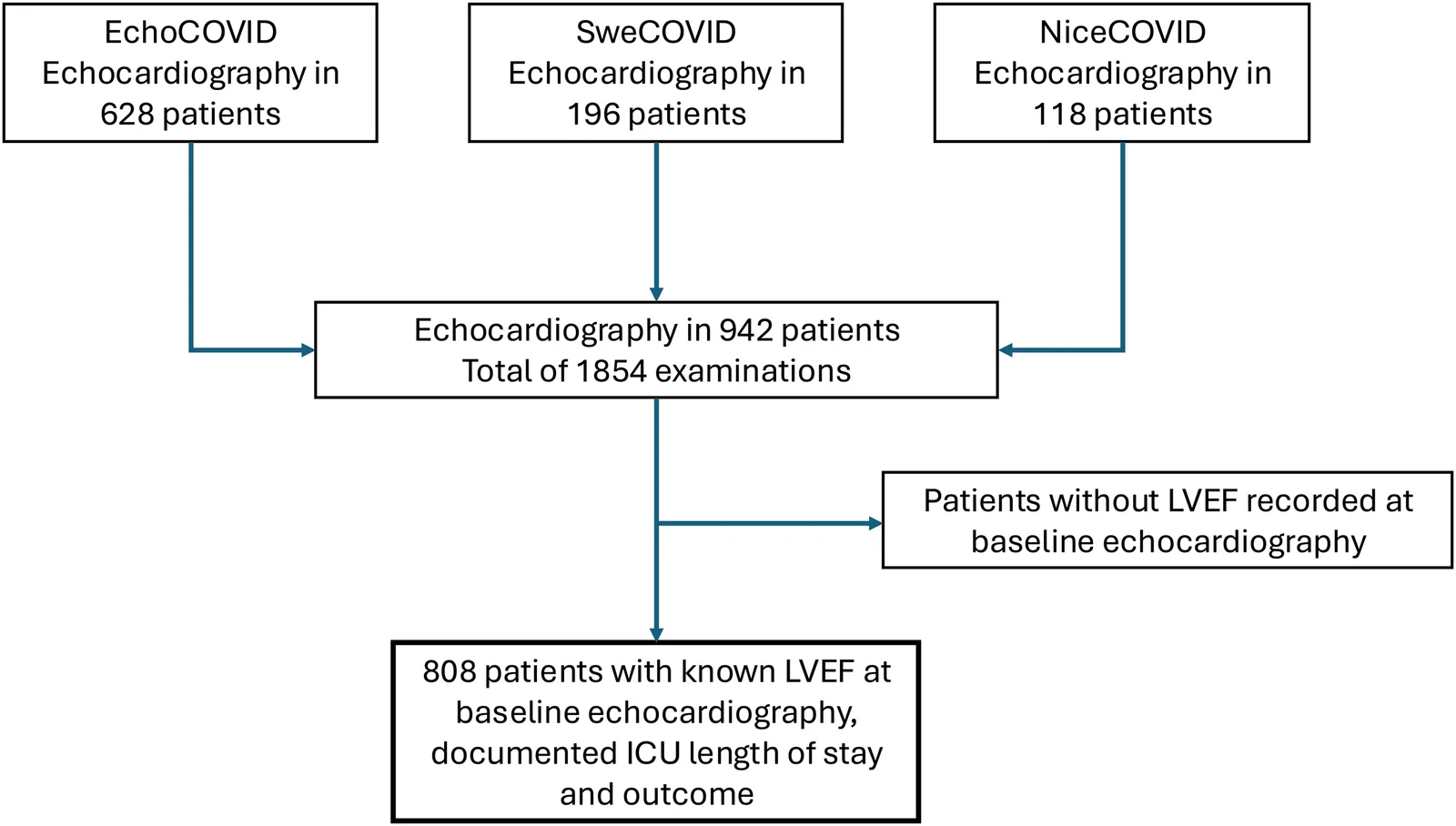
Cohort flow chart.

Demographic, anthropometric and haemodynamic data from ICU admission to discharge were extracted from each database. A complete list of variables available in all three repositories can be found as a Supplementary file. TTE and TEE examinations of patients from ICU admission to discharge or death during ICU stay were included. Images were interpreted off-line by international experts in the field with European Diploma in Advanced Critical Care Echocardiography or equivalent qualifications and experience. Left ventricular function was defined as abnormal (LVEF ≤50%), normal (LVEF 51–70%) and hyperdynamic (LVEF > 70%) by Simpson’s method or visual assessment. RV function was defined as the presence or absence of ACP defined as the right to left end-diastolic area ratio of >0.6 and septal dyskinesia. We also collected data on forward flow i.e. the velocity time integral at the left ventricular outflow tract (LVOT VTI). We compared the fit of models specifying LVEF as a continuous linear term, a nonlinear restricted cubic spline function, or a categorical variable. There was no evidence of non-linearity (ANOVA p = 0.48) with the Akaike Information Criterion differed by 2.3 units across the models, indicating statistically equivalent performance across all three specifications. Because the continuous and spline models did not meaningfully outperform the categorical, we retained the categorical model to maximize clinical interpretability, comparison with previous literature and preserve actionable risk thresholds.

We evaluated the association between ICU mortality and time-varying LVEF categories using a mixed-effects Cox proportional hazards model incorporating time-dependent covariates. Follow-up was structured into interval-based counting processes. Time-dependent clinical and echocardiographic variables were updated at each assessment using a last-observation-carried-forward approach. To avoid immortal time bias, follow-up began at the time of the initial echocardiogram, and prior ICU intervals were excluded. We applied a systematic imputation strategy: time‑fixed variables (age, body mass index (BMI)) were imputed using country‑specific medians, while cardiovascular history (defined as one or more of diabetes, hypertension or cardiac failure) was retained as a separate "Unknown" category. Time‑varying covariates (treatments, haemodynamics, echocardiographic parameters) were carried forward to the next measurement occasion. Categorical time‑varying variables with missing values (vasopressor, IMV, etc.) were coded as "Unknown". The full Cox model adjusted for baseline characteristics (age, sex, BMI), cardiovascular history (one or more of diabetes, hypertension or cardiac failure); and the following time-varying covariates: vasopressor treatment, invasive mechanical ventilation, heart rate, diastolic arterial pressure, left ventricular outflow tract velocity time integral (LVOT VTI) dichotomized to > and ≤14 cm, acute cor pulmonale, E/A-ratio, PaO_2_/FiO_2_ ratio. To account for possible country level differences and repeated measurements within patients, we specified patients as nested within countries, using the “coxme package” in R. The likelihood ratio test indicated significantly improved model fit when country was added (p < 0.001), we therefore retained the nested country–patient random-effects structure in all subsequent models. Model selection followed backward stepwise elimination based on Wald p-values, clinical relevance and likelihood ratio testing. Because the SweCOVID and Nice databases only included a maximum 3 echocardiograms by design a sensitivity analysis was conducted including only the first 3 echocardiograms. All analyses were conducted in R.

## Results

After removal of duplicates, 942 unique patients were available of analysis - 628, 196, 118 patients respectively from the EchoCOVID, SweCOVID and Nice databases, of which 808 had available LVEF data on the first echocardiographic examination. The total number of included patients with echocardiographic examinations and echocardiographic variables are given in the flowchart. Fig. 1 shows the study flow chart describing the study cohort.

The minimum dataset was based on the 808 patients that had available data for LVEF (main exposure) at baseline. The number of patients with available echocardiographic data during the subsequent second to fifth echocardiographic examinations were 384, 234, 89 and 48. Some patients had up to 12 echocardiographic examinations during ICU stay, but the number of patients with >5 examinations were very few and these echocardiograms were not included in the analyses. The first echocardiographic examination was conducted on median day 1 [1,3], the second examination on day 4 [4,6], the third on day 8 [7,11].

Table 1 shows the population characteristics at the first echocardiographic examination. Supplementary Table 1 presents population characteristics in the three different cohorts. Out of the 808 patients, 304 (38%) died in the ICU. Mortality differed across the cohorts, from 13% (SweCOVID), 25% (Nice) to 35% (EchoCOVID).

**Table 1:** Population characteristics.

Patient characteristics at baseline echocardiography n = 808		Number missing
Age [Median (25,75%)]	65 (57,73)	75
Sex [Male: number (%)]	563 (70%)	0
BMI [Median (25,75%)]	28.4 (25.5, 33.2)	28
Hypertension [number (%)]	443 (55%)	8
Diabetes [number (%)]	235 (29%)	5
Cardiac Failure [number (%)]	194 (14%)	62
Pre-existing cardiovascular disease [number (%)]	475 (64%)	66
Severity of illness [Median (25,75%)]		
EchoCOVID	NA	0
Nice (SAPS II)	44 (31, 65)	7
Linköping (SAPS III)	52 (47, 62)	
Heart rate [bpm]	83 (70,99)	21
Mean Arterial Pressure [mmHg]	82 (72,94)	32
PaO_2_/FiO_2_ [mmHg (25,75%)]	120 (86,173)	30
LVEF [Median (25,75%)]	59 (50,65)	0
LVEF normal [number (%)]	458 (57%)	0
LVEF abnormal [number (%)]	214 (26%)	0
LVEF hyperdynamic [number (%)]	136 (17%)	0
LVOT VTI [Median (25,75%)]	20.0 (17.0, 23.2)	85
Acute cor pulmonale [number %]	83 (11%)	46
Invasive mechanical ventilation [number (%)]	508 (64.8%)	24
ICU length of stay [Median (25,75%)]	18 (9,39)	0
ICU mortality [number (%)]	304 (38%)	0

BMI = body mass index; SAPS = Simplified Acute Physiology Score; LVEF = left ventricular ejection fraction; VTI = velocity time integral at the left ventricular outflow tract. LVEF normal is defined as LVEF 51–70%. LVEF abnormal is defined as ≤50%, LVEF hyperdynamic is defined as >70%.

A total of 1614 echocardiographic examinations were analysed in the 808 patients. LVEF was normal in 939 (58.2%), abnormal in 366 (22.7%) and hyperdynamic in 309 (19.1%) during the whole observational period. Among the 808 patients, abnormal LVEF was observed in 26.5% and hyperkinesia in 16.7% at the first echocardiographic examination. The prevalence of abnormal LVEF was highest on the first examination (26.5%), then decreased in subsequent examinations to 16.7% at the fifth echocardiographic examination. The prevalence of hyperdynamic LVEF phenotype increased during ICU stay, then returned to baseline levels by the fifth examination. The proportion of normal LVEF was highest at the last (5^th^) examination (Fig. 2). Among all examinations severely impaired LVEF (≤30%) was found in 4.5% overall and 2.6% on the first echocardiographic examination.

**Fig. 2:**
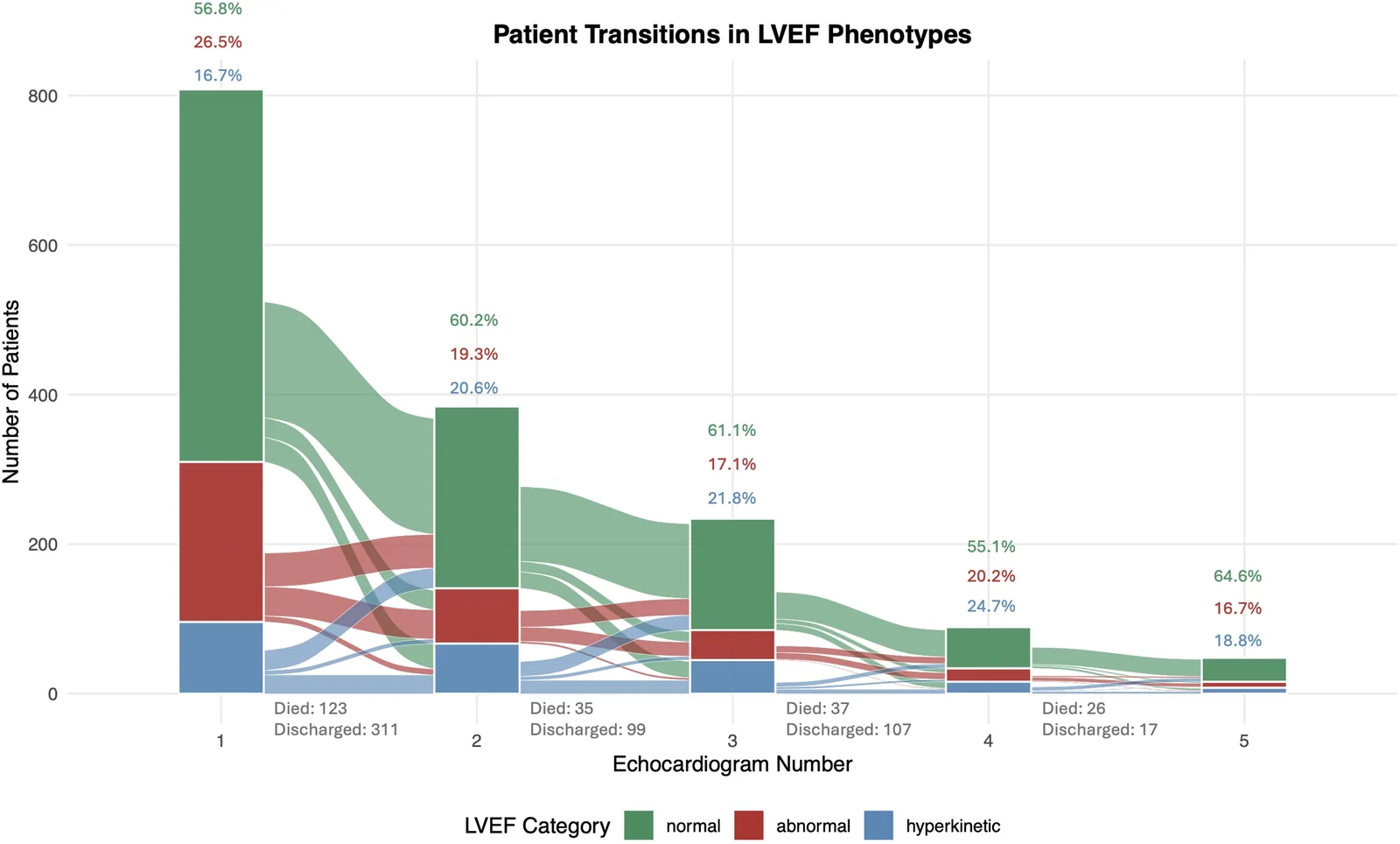
Time varying incidence LVEF phenotypes over the first five echocardiographic examinations stratified by abnormal (≤50%), normal (51–70%) and hyperdynamic (>70%).

Fig. 3 shows the numbers of observations with the different LVEF phenotypes (normal, hypodynamic and hyperdynamic) concurrently with acute cor pulmonale and high or low LVOT VTI. There were 711 unique patients with 1235 echocardiographic examinations with simultaneously documented LVEF, ACP and LVOT VTI. Note that a different phenotype might have occurred in another echocardiographic examination for a given patient.

**Fig. 3:**
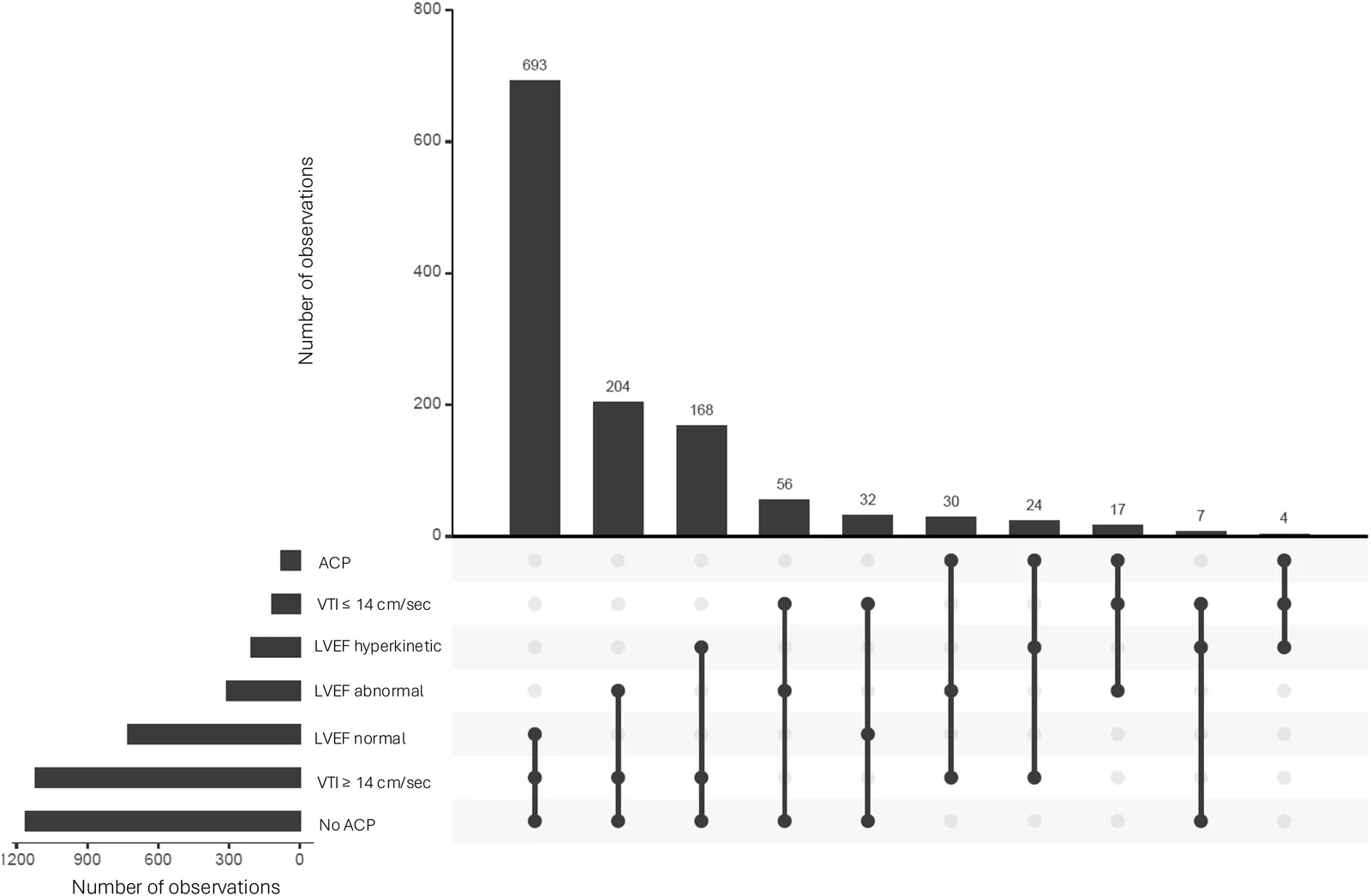
Upset plot showing the numbers of observations (echocardiographies) with the different LVEF phenotypes (normal, hypokinetic or hyperkinetic) and simultaneous acute cor pulmonale (yes or no) and LVOT VTI (low if ≤14 and high if >14 cm). The horizontal bars show the distribution of echocardiography phenotypes of LV involvement, ACP and VTI. The vertical bars represent the number of observations with different combinations of echocardiography phenotypes of LV involvement, ACP and VTI. Dots represent the echocardiography phenotypes, and lines connecting the dots represent the combinations of phenotypes.

After backward stepwise elimination age, cardiovascular history, vasopressor treatment, PaO_2_/FiO_2_ ratio and acute cor pulmonale were retained. Time-varying LVEF was not an independent predictor of ICU mortality in the Cox regression model. When modelled as time-varying covariates, the presence of ACP was an independent predictor of outcome, as was the PaO_2_/FiO_2_ ratio during ICU admission. The presence of previous cardiovascular disease was also associated with ICU mortality (Table 2). The full Cox regression model is available in the Supplementary material (Supplementary Table 2).

**Table 2:** Cox regression model with left ventricular ejection fraction (LVEF) phenotypes as a time-varying exposure.

Variable	aHR	2.5%	97.5%	p-Value
Age	1.026	1.012	1.04	<0.001
Previous cardiovascular disease	0.717	0.526	0.977	0.035
Vasopressor	1.168	0.854	1.597	0.332
PaO_2_/FiO_2_	0.993	0.992	0.995	<0.001
LVEF class (ref: normal)				
Abnormal LVEF	1.174	0.901	1.531	0.236
Hyperdynamic LVEF	1.041	0.785	1.379	0.782
Acute cor pulmonale	1.653	1.228	2.225	0.001

A sensitivity analysis using only the first 3 echocardiographic examinations is provided in Supplementary Table 3. This analysis did not demonstrate any effect not seen in the original 5-echo model.

## Discussion

In this cohort of critically ill patients with COVID-19, LVEF modelled as a time-varying exposure was not independently associated with ICU mortality. These findings extend previous knowledge by incorporating longitudinal changes in LV function during ICU stay and adjusting for forward flow (LVOT VTI) and ACP, which has previously been identified as an independent predictor of mortality. Advanced age, pre-existing cardiovascular disease, and ACP emerged as the strongest determinants of ICU mortality.

Abnormal LV systolic function declined over time, suggesting partial reversibility, while hyperdynamic LV function increased during ICU stay before returning to baseline by the fifth examination. These dynamic patterns indicate that LV performance in severe COVID-19 is not static but evolves in response to disease progression and treatment. Reliance on snapshot examinations may obscure clinically relevant myocardial dysfunction present in a sizeable subgroup. Despite accounting for temporal differences, no relationship was demonstrable between LVEF subgroups and ICU mortality.

The prognostic significance of LV dysfunction in COVID-19 has been inconsistent across studies. In our previous analysis of the EchoCOVID cohort, visually assessed LV systolic dysfunction was associated with in-hospital mortality (OR = 1.52 [1.04, 2.23]), but this relationship was no longer significant after adjustment for age [6]. Conversely, another large multicenter study demonstrated that severely reduced LVEF was independently associated with ICU mortality [14]. Our findings are in line previous studies demonstrating mildly reduced LVEF within 24 h of ICU admission in patients with COVID-19 disease [12,13,15], although earlier studies generally do not account for temporal changes.

Severe LV hypokinesia (LVEF < 30%) was uncommon, affecting 4.5% of patients, consistent with previous estimates of 4.7%–8.7% [6,14,16], but this subgroup was too small for robust statistical analysis. At the opposite end of the spectrum, hyperdynamic LV function (LVEF > 70%) was observed in approximately one-fifth of examinations, similar to previous findings in septic shock [17], with prevalence increasing during ICU stay. Although fluid balance data were unavailable, this may reflect restrictive fluid therapy management to limit pulmonary oedema. The use of vasopressors and inotropes may also explain why the prevalence of hyperdynamic LV function increases during ICU stay. Notably, unlike in septic shock, hyperdynamic LV function was not associated with increased ICU mortality in this cohort [18,19], potentially explained by pathophysiological differences between septic shock and COVID-19, where the latter primarily causes respiratory symptoms and not necessarily systemic vasodilation [20].

Right ventricular function has been more extensively studied than LV function in critically ill COVID-19 patients [4,7,8,21]. In particular, ACP has emerged as a marker of poor prognosis across several cohorts, likely representing a physiologically extreme stage of RV adaptation to increased afterload and that evolves dynamically during the course of COVID-19 related Acute Respiratory Distress Syndrome [4,5,7,8,22]. In our cohort, ACP but not invasive mechanical ventilation, was associated with ICU mortality. Whereas studies addressing LV function have largely relied on single, early “snapshot” examinations, repeated assessment of RV function has been undertaken in at least two major investigations. Jozwiak et al. demonstrated that the combination of RV dysfunction with RV dilation or ACP showed a potential association with 28-day mortality [22]. Development of RV failure during ICU stay was a strong independent predictor of 90-day mortality [8] and, in the largest study to date, the EchoCOVID group reported that critically ill patients may transition between different RV phenotypes over time; those with demonstrable ACP on the final echocardiographic examination during ICU stay had the highest risk of death [7]. However, the assessment of ACP was not adjusted for concomitant LV dysfunction, which may be relevant given the substantial contribution of LV contraction to RV performance through ventricular interdependence. Accounting for LV function could therefore provide a more nuanced evaluation of the independent prognostic impact of RV pathology [23].

Overall, our findings suggest that LV systolic dysfunction measured as LVEF in severe COVID-19 is common but frequently transient and does not independently drive mortality once key haemodynamic factors such as forward flow and ACP are accounted for. Whether other more sensitive and less load dependent indicators of LV systolic function such as global longitudinal strain and mitral annular plane systolic excursion may provide more nuanced information remains underinvestigated.

## Limitations

Several limitations should be acknowledged. Although the Linköping and Nice cohorts enrolled consecutive patients, the EchoCOVID cohort included only patients in whom echocardiography was indicated clinically, raising the possibility of selection bias. This may explain the lower prevalence of ACP in the present study, compared to that previously reported for the EchoCOVID. We did not have data on presence of pulmonary embolism and the information about vasopressor and inotropic treatment was sparse. Access to more information on these variables could have enabled a more nuanced analysis. There was a considerable drop-out throughout the study, however it was not unexpected. Several factors can explain this: mortality, ICU discharge and for the ECHO-COVID study no clinical indication for further echocardiographic examinations. Since the Linköping and Nice cohorts included up to three echocardiographic examinations by study design, all patients lack subsequent echocardiographic examinations and would thus be classified as drop-outs. However, the sensitivity analysis restricted to the first three echocardiographic examinations produced consistent results.

The confidence intervals around our primary hazard ratio estimates are relatively wide and compatible with both a modest protective and a modest harmful association between LVEF and mortality. Our findings should therefore be considered inconclusive on this point rather than definitive evidence of no association.

We could not study LV function in a granular way: more sensitive, indicators of LV systolic dysfunction such as LV global longitudinal strain and mitral annular plane systolic excursion [[24], [25], [26]] were unavailable in the EchoCOVID database. These may have provided a more nuanced assessment, especially among patients with subclinical systolic dysfunction. Finally, we assessed only ICU mortality, but other clinical outcomes such as acute myocardial injury and organ support duration would be relevant lines of investigation.

## Conclusions

In this large cohort of critically ill COVID-19 patients, decreased and hyperdynamic LVEF were common at baseline, and the proportion of patients with normal LVEF increased during ICU stay. Modelled as a time-varying exposure and adjusted for forward flow, RV function, and key clinical covariates, neither abnormal nor hyperdynamic LVEF was independently associated with ICU mortality, whereas ACP remained a strong independent predictor. These findings suggest that although LV systolic abnormalities are frequent, their prognostic value for ICU mortality in severe COVID-19 may be limited, and more sensitive measures of LV function, myocardial injury biomarkers, and broader clinical outcomes are needed to clarify the role of cardiac involvement in this setting.

## CRediT authorship contribution statement

MSC: MSC designed the study. MS; AVB and SH created the Echo-COVID database. MJ was responsible for the Nice cohort. MSC; SJ; ZH; HD and CJ collected and cleaned data for the SweCOVID cohort. SJ and HA combined the datasets and cleaned data. HZ; MSC and SJ performed statistical analyses. MSC and SJ drafted the manuscript. All authors read and approved the final manuscript.

## Ethics approval and consent to participate

Ethical approval was given by each local ethics committee (EchoCOVID, CE SLRF 20-40; Nice, R04-0223313140420; SweCOVID, Dnr 2020-01884).

No consent was required due to the retrospective and observational method of the study.

## Consent for publication

Not applicable.

## Funding

Open access funding provided by Linkoping University. This study was funded by grants from the Region Ostergotland ALF grants and Linkoping University (LiO3032008, LiO935252). The funding bodies had no role in the design of the study and collection, analysis, and interpretation of data and in writing the manuscript.

## Data availability

The data from this study will be made available after publication, upon application to the corresponding author and within the terms of the Global Data Protection Regulation and the Swedish Patient Data Law (2008:355). To avoid the possibility of identifying individual cases, detailed data are not given in the paper but may be requested from the corresponding author.

## Declaration of competing interest

AVB declares research grant from GSK and Air Liquid companies. DDB declares honoraria from Philips company for lecture and honoraria for consulting from Edwards Lifesciences, Pfizer and Baxter companies. MSC declares honoraria and consulting fees from Edwards Lifesciences, Laboratorie Agguetant, AOP Health, Philips Healthcare, The other authors declare that they have no conflict of interest.
