# Supplementary material for: The effect of time-varying left ventricular ejection fraction on clinical outcome in critically ill COVID-19 patients
Source: Ann Intensive Care. 2026 Sep 29;16:100171. doi: 10.1016/j.aicoj.2026.100171 (PMC13677176; doi:10.1016/j.aicoj.2026.100171)
Supplement: Supplementary file 1 [file mmc2.pdf]

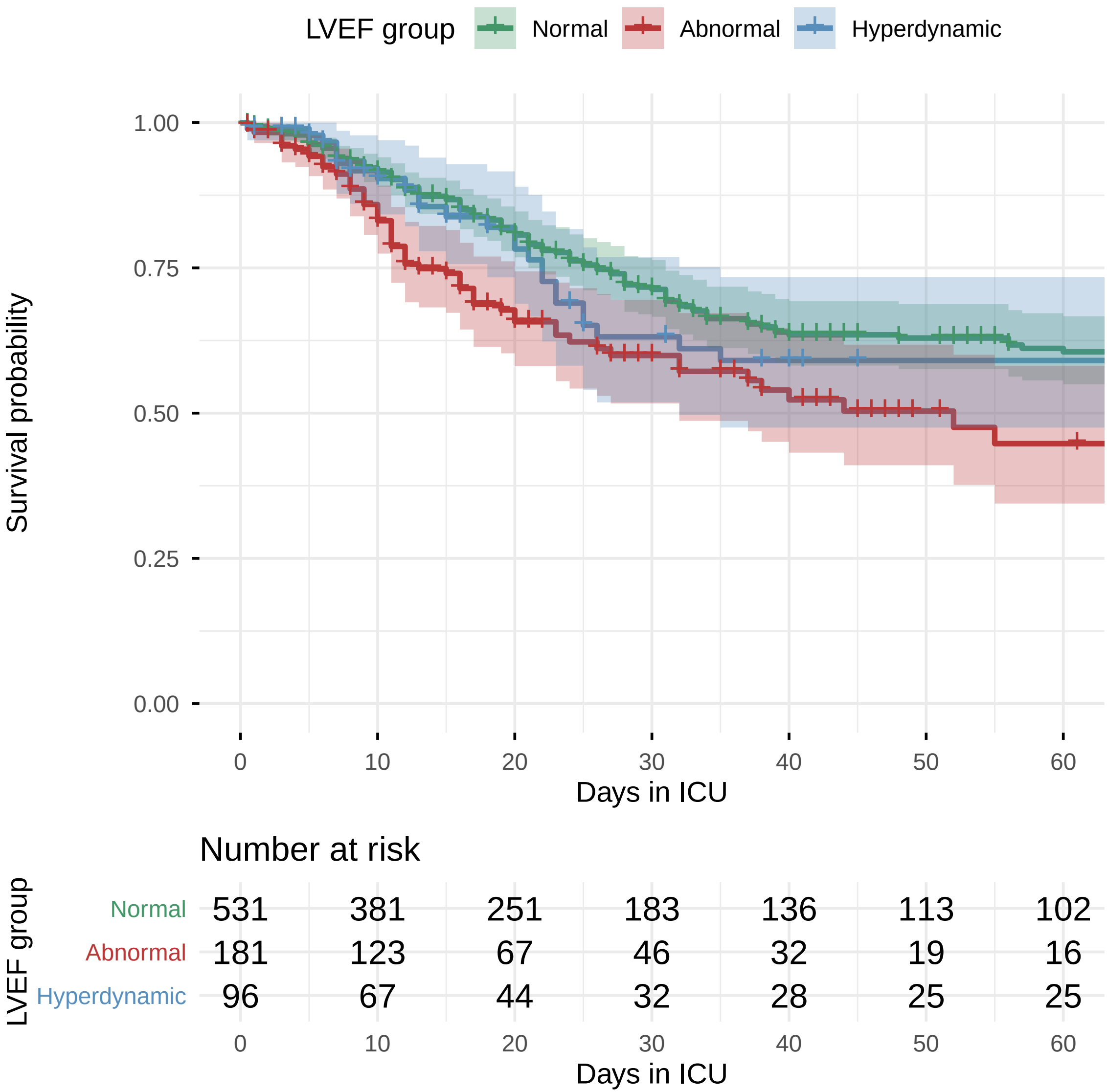
LVEF group
Normal
Abnormal
Hyperdynamic
Survival probability
1.00
0.75
0.50
0.25
0.00
0
10
20
30
40
50
60
Days in ICU
Number at risk
LVEF group
Normal 531 381 251 183 136 113 102
Abnormal 181 123 67 46 32 19 16
Hyperdynamic 96 67 44 32 28 25 25
0
10
20
30
40
50
60
Days in ICU
