# Supplementary material for: The effect of time-varying left ventricular ejection fraction on clinical outcome in critically ill COVID-19 patients
Source: Ann Intensive Care. 2026 Sep 29;16:100171. doi: 10.1016/j.aicoj.2026.100171 (PMC13677176; doi:10.1016/j.aicoj.2026.100171)
Supplement: Supplementary file 2 [file mmc1.docx]

**Supplementary table 1, Population characteristics split by repository**

| **Patient characteristics at baseline echocardiography n=808** | **EchoCOVID**  *n =* 575 | **SweCOVID**  *n =* 115 | **NiceCOVID**  *n =* 118 | **p-value** |
| --- | --- | --- | --- | --- |
| Age [Median (25,75%)] | 66 (57,73) | 61 (53,72.5) | 64.7 (59.7,72.8) | 0.263 |
| Sex [Male: number (%)] | 411 (72%) | 68 (59%) | 84 (71%) | 0.029* |
| BMI [Median (25,75%)] | 28.4 (25.4,33.2) | 29.7 (25.3,34.7) | 28.1 (26,31) | 0.274 |
| Hypertension [number (%)] | 331 (58%) | 54 (47%) | 58 (49%) | 0.034* |
| Diabetes [number (%)] | 170 (30%) | 30 (26%) | 35 (30%) | 0.756 |
| Cardiac Failure [number (%)] | 91 (16%) | 9 (8%) | 4 (3%) | <0.001* |
| Pre-existing cardiovascular disease [number (%)] | 347 (60%) | 61 (53%) | 67 (57%) | 0.002* |
| Heart rate [bpm] | 87.5 (74,103 | 68 (56,83) | 76.5 (68-86) | <0.001* |
| Mean Arterial Pressure [mmHg] | 83 (72,95) | 80 (72.5,87) | 80 (75-92.5) | 0.043* |
| PaO_2_/FiO_2_ [mmHg (25,75%)] | 116 (85,162.5) | 152.3 (104.5,205.1) | 115 (79.6,189.2) | <0.001* |
| LVEF [Median (25,75%)] | 60 (49,65) | 55 (50,61.5) | 62 (55-68.7) | <0.001* |
| LVEF normal [number (%)] | 340 (59%) | 81 (70%) | 77 (65%) | 0.051 |
| LVEF abnormal [number (%)] | 163 (28%) | 32 (28%) | 19 (16%) | 0.021* |
| LVEF hyperdynamic [number (%)] | 72 (13%) | 2 (2%) | 22 (19%) | <0.001* |
| LVOT VTI [Median (25,75%)] | 19.3 (16.7,22,5) | 21 (17.5,24.7) | 21.9 (18.1,24.4) | <0.001* |
| Acute cor pulmonale [number %] | 78 (14%) | 3 (3%) | 2 (2%) | <0.001* |
| Invasive mechanical ventilation [number (%)] | 393 (68%) | 64 (60%) | 62 (53%) | 0.002* |
| ICU length of stay [Median (25,75%)] | 16 (9,28) | 9 (3.5,18) | 12.5 (6.25,25) | <0.001* |
| ICU mortality [number (%)] | 201 (35%) | 15 (13%) | 30 (25%) | <0.001* |

BMI = body mass index, LVEF = = left ventricular ejection fraction, LVOT VTI = left ventricular outflow tract velocity time integral, ICU = intensive care unit. LVEF normal is defined as LVEF 51-70%. LVEF abnormal is defined as ≤50%, LVEF hyperdynamic is defined as >70%.

**Supplementary table 2, Full Cox regression model with left ventricular ejection fraction (LVEF) phenotypes as a time-varying exposure.**

| **Variable** | **aHR** | **2.5%** | **97.5%** | **p-value** |
| --- | --- | --- | --- | --- |
| Age | 1.003 | 1.018 | 1.049 | <0.001 |
| Sex, female | 1.055 | 0.769 | 1.446 | 0.724 |
| BMI | 1.001 | 0.976 | 1.027 | 0.929 |
| Previous cardiovascular disease | 0.717 | 0.526 | 0.977 | 0.035 |
| Vasopressor | 0.958 | 0.669 | 1.373 | 0.815 |
| Invasive mechanical ventilation | 1.154 | 0.823 | 1.62 | 0.406 |
| PaO_2_/FiO_2_ | 0.994 | 0.992 | 0.995 | <0.001 |
| Heart rate | 1.005 | 0.999 | 1.012 | 0.075 |
| Diastolic arterial pressure | 0.997 | 0.987 | 1.007 | 0.581 |
| LVEF class (ref: normal)  Abnormal LVEF  Hyperdynanmic LVEF | 1.213  1.066 | 0.901  0.795 | 1.633  1.429 | 0.202  0.669 |
| Acute cor pulmonale | 1.722 | 1.244 | 2.384 | 0.001 |
| LVOT VTI | 1.01 | 0.987 | 1.033 | 0.400 |
| E/A-ratio | 1.384 | 1.009 | 1.9 | 0.044 |

BMI = body mass index, LVEF = left ventricular ejection fraction, LVOT VTI = left ventricular outflow tract velocity time integral. LVEF normal is defined as LVEF 51-70%. LVEF abnormal is defined as ≤50%, LVEF hyperdynamic is defined as >70%.

**Supplementary table 3, Cox regression model with left ventricular ejection fraction (LVEF) phenotypes as a time-varying exposure, limited to first 3 echocardiographic examinations**

| **Variable** | **aHR** | **2.5%** | **97.5%** | **p-value** |
| --- | --- | --- | --- | --- |
| Age | 1.034 | 1.02 | 1.048 | <0.001 |
| Sex, female | 1.063 | 0.803 | 1.407 | 0.671 |
| BMI | 1.006 | 0.984 | 1.029 | 0.598 |
| Previous cardiovascular disease | 0.69 | 0.524 | 0.908 | 0.008 |
| Vasopressor | 0.059 | 0.681 | 1.351 | 0.81 |
| Invasive mechanical ventilation | 0.986 | 0.715 | 1.36 | 0.93 |
| PaO_2_/FiO_2_ | 0.995 | 0.994 | 0.997 | <0.001 |
| Heart rate | 1.004 | 0.997 | 1.01 | 0.262 |
| Diastolic arterial pressure | 1.004 | 0.993 | 1.014 | 0.483 |
| LVEF class (ref: normal)  Abnormal LVEF  Hyperdynanmic LVEF | 1.282  0.999 | 0.95  0.737 | 1.73  1.354 | 0.104  0.992 |
| Acute cor pulmonale | 1.448 | 1.012 | 2.071 | 0.043 |
| LVOT VTI | 1.019 | 0.996 | 1.042 | 0.112 |
| E/A-ratio | 1.552 | 1.142 | 2.111 | 0.005 |

BMI = body mass index, LVEF = left ventricular ejection fraction, LVOT VTI = left ventricular outflow tract velocity time integral. LVEF normal is defined as LVEF 51-70%. LVEF abnormal is defined as ≤50%, LVEF hyperdynamic is defined as >70%.
