# Supplementary material for: The effect of time-varying left ventricular ejection fraction on clinical outcome in critically ill COVID-19 patients
Source: Ann Intensive Care. 2026 Sep 29;16:100171. doi: 10.1016/j.aicoj.2026.100171 (PMC13677176; doi:10.1016/j.aicoj.2026.100171)
Supplement: Supplementary file 4 [file mmc4.docx]

**List of variables**

| *Baseline and outcome variables* |  |
| --- | --- |
| record_id | Unique subject number |
| Country | Country where subject is treated |
| EchoNo | Number of echocardiography (1-5) |
| StudyDate | Date of echocardiography |
| Echo_DayInICU | Day in ICU echocardiography is performed |
| Date_1st_clin_signs | Date of first reported signs of COVID-19 |
| Date_ICU_adm | Date of ICU admission |
| Date_ICU_dchrg | Date of ICU discharge |
| Date_hosp_dchrg | Date of hospital discharge |
| ICUStatus | Survived or died in ICU |
| HosStatus | Survived or died in hospital |
| Sex | Gender (female/male) |
| Age | Age in years |
| Weight | Weight in kilograms |
| Height | Height in centimeters |
| BMI | Body mass index (kg/m^2^) |
| Cardiac failure | Presence of cardiac failure diagnosis |
| Hypertension | Presence of hypertension diagnosis |
| Diabetes | Presence of diabetes diagnosis |
| *Echocardiography parameters* |  |
| Echo_type | Type och echocardiography: TTE (transthoracic) or TEE (transesophageal) |
| Cardiac_rythm | Rythm: sinus, atrial fibrillation or flutter, pacemaker |
| LV_function_vis | Visual assessment of left ventricular function: hyperkintetic, normal, moderately depressed, severely depressed |
| LVEF | Left ventricular ejection fraction in percentage |
| SWMA | Segmental wall motion abnormality: absent or present |
| LV_size_vis | Visual assessment of left ventricular size: reduced, not dilated, moderately dilated, severely dilated |
| LVEDD | Left ventricular end diastolic diameter in millimeters |
| EA_ratio | E/A-ratio |
| LV_gls | Left ventricular global longitudinal strain in percentage |
| VTIaorta | Left ventricular outflow tract velocity time integral in centimeters |
| RV_function_vis | Visual assessment of right ventricular function: hyperkinetic, normal, moderately depressed, severely depressed |
| TAPSE_mm | Tricuspid annular plane systolic excursion in millimeters |
| RV_FAC% | Right ventricular fractional area change in percentage |
| PSM | Paradoxal septal movement: yes or no |
| RV_size_vis | Visual assessment of right ventricular size: reduced, not dilated, moderately dilated, severely dilated |
| RV:LV_EDA | Ratio between right ventricular and left ventricular end diastolic area |
| TRVmax | Squared maximum tricuspid regurgitant velocity in meters per second |
| IVC_diam | Inferior vena cava diameter: small, normal, dilated |
| Pericard_eff | Pericardial effusion: yes or no |
| PFO | Patent foramen ovale: yes or no |
| *Clinical parameters during echo* |  |
| Drug_1 | First recorded vasopressor/inotropic drug |
| Drug_2 | Second recorded vasopressor/inotropic drug |
| Drug_3 | Third recorded vasopressor/inotropic drug |
| Vent_mode | Mechanically ventilated or spontaneously breathing |
| FiO2 | Fraction inhaled oxygen in percentage |
| RR | Respiratory rate |
| TV | Tidal volume in milliliters |
| PEEP | Peak expiratory pressure in centimeters of water |
| PaO2 | Partial pressure of oxygen in mmHg |
| PaCO2 | Partial pressure of carbon dioxide in mmHg |
| pH | pH in blood |
| Base_deficit | Base deficit in blood in mEq/l |
| Lactate | Lactate in blood in mmol/l |
| HR | Heart rate |
| SAP | Systolic arterial pressure |
| DAP | Diastolic blood pressure |
| MAP | Mean arterial pressure |
| CVP | Central venous pressure |
